# Gamification to Support Adherence to a Therapeutic Ambylopia Treatment for Children: Retrospective Study Using a Focal Ambient Visual Acuity Stimulation Game

**DOI:** 10.2196/32282

**Published:** 2023-02-01

**Authors:** Catheline Bocqué, Jingyun Wang, Annekatrin Rickmann, Henrike Julich-Haertel, Uwe Kaempf, Kai Januschowski

**Affiliations:** 1 Klaus Heimann Eye Research Institute Sulzbach Germany; 2 State University of New York College of Optometry New York, NY United States; 3 Caterna Vision GmbH Potsdam Germany; 4 Centre for Ophthalmology University Eye Hospital Eberhard-Karls-University Tuebingen Germany; 5 Mt St Peter Eyeclinic Trier Germany

**Keywords:** amblyopia, children, compliance, adherence, occlusion, patching, therapeutic game, FAVAS

## Abstract

**Background:**

The gold standard treatment for amblyopia is patching the better eye. Improvement of visual acuity in the amblyopic eye is significantly impacted by the adherence to the patching therapy. It is known that the overall adherence is rather low.

**Objective:**

This retrospective study evaluated whether an updated version of attention-binding digital therapeutic games based on the principle of focal ambient visual acuity stimulation (FAVAS) would result in improved patient adherence in 4- to 16-year-old patients with amblyopia associated with anisometropia or strabismus.

**Methods:**

We analyzed electronically pseudonymized recorded data from patients treated with occlusion therapy and FAVAS therapeutic games. One group used an older version (2015) and the other group used a newer version (2020) that provided more attractive therapeutic games with tablet computer functionality. Objective adherence was calculated by comparing the number of minutes using the therapeutic games as monitored in the automatized logbook versus the prescribed number of minutes for using the games.

**Results:**

Children in group 2015 (n=138) spent on average 2009.3 (SD 1372.1; range 36-5556) minutes using FAVAS; children in group 2020 (n=129) spent on average 2651.2 (SD 1557.1; range 38-5672) minutes using the newer version. Group 2020 spent on average 641.9 more minutes on FAVAS than group 2015 (*t*_255.49_=3.56, *P*<.001, *d*=0.45; 95% CI 0.69-0.20). Although patient adherence was very variable, compared to the 55.0% (SD 29.4%) in group 2015, it significantly improved up to 68.5% (SD 33.7%) in group 2020 (*t*_254.3__8_=3.48, *P*=.001, *d*=0.44; 95% CI 0.68-0.19).

**Conclusions:**

FAVAS 2020, with improved gamification aspect as well as tablet computer functionality, increased adherence significantly compared to the earlier version of FAVAS 2015, indicating that FAVAS 2020 could be an effective approach to support adherence to amblyopia treatment.

**Trial Registration:**

German Clinical Trials Register (DRKS) DRKS00017633; https://drks.de/search/de/trial/DRKS00017633

## Introduction

Unilateral amblyopia is a developmental disorder resulting in degraded visual acuity in 1 eye. During the developmental phase of vision, degraded stimulation by the weaker eye leads to the underdevelopment of the corresponding cortical visual areas [1]. Amblyopia is associated with poor binocular visual experience in children and has a lasting effect on the individuals’ quality of life, while children with amblyopia are impacted in their daily activities and future job selection [2]. It also increases the risk of severe trauma for the fellow sound eye [3]. Occlusion therapy with patching, after optical adaptation with binocular eyeglass correction, has been the gold standard therapeutic approach for forcing the visual development of the amblyopic weaker eye by an input deprivation of the other sound eye since Sattler [4]. However, by patching, a high rate of patients (approximately 25% to 30%) do not show a full recovery of visual function, and some of those patients even show further worsening in visual function [5-8]. Visual acuity improvement in the amblyopic eye is significantly impacted by adherence to patching therapy [9]. For a long time, a system of monocular and binocular visual exercises and stimulation methods (pleoptics and orthoptics) in support of the standard occlusion treatment has been developed, but only with limited success [10-12]. Some perceptual learning treatments of the last few years have been monocular training with grating contrast detection tasks or viewing action movies and video games. Some binocular treatments work by presenting dichoptic, high-contrast stimuli to the amblyopic eye and low-contrast stimuli to the other eye during video games. Other binocular treatments do not involve contrast balancing but instead present dichoptic videos or video games with the background presented to both eyes and foreground elements presented only to the amblyopic eye [13,14].

This study explores the a priori hypothesis that the updated version of monocular focal ambient visual acuity stimulation (FAVAS) therapeutic games has improved patient adherence*.* To improve adherence to patching therapy, gamification of therapy could encourage the patient to actively use the amblyopic eye. FAVAS therapeutic games are an innovative digital therapeutic designed as a supplementary treatment to patching. A customized moving ambient sinusoidal wave pattern (moving gratings) is presented in the background of focal attention-binding digital therapeutic games, stimulating cortical areas to activate the central perceptive activity of the amblyopic eye again and thus improving visual acuity [15].

At the same time, ambient stimulation is provided in the game’s background by a drifting sinusoidal contrast-modulated grating pattern of constant spatial and temporal frequency. Due to its periodicity, the drifting grating stimulus is assumed to induce resonance within and between filter systems of band-pass selective neuronal transmission channels [15-17]. The stimulus is a drifting sinusoidal grating with a spatial frequency of 0.3 and a temporal frequency of 1 cycle per second, reciprocally coordinated with each other to produce a drift of 0.33 degrees per second. The customized pattern takes the axis of astigmatism into account, there are 3 groups of best-corrected visual acuity (BCVA; <0.2, 0.2-0.5, >0.5), and with strabismus there is a circular stimulation. The software-implemented exercises for our visual training are based on a specially developed FAVAS. In the foreground of the screen, a focal computer game demands sensory-motor coordination, visual fixation performance, and adherence from the children. Thus, the gaming activity serves mainly for attention binding, which has been previously proven to be a decisive factor for the success of visual-training exercises. Previously, Kämpf et al [16] showed that FAVAS had a promising effect on the visual acuity of amblyopic eyes in a specific way.

**Figure 1:**
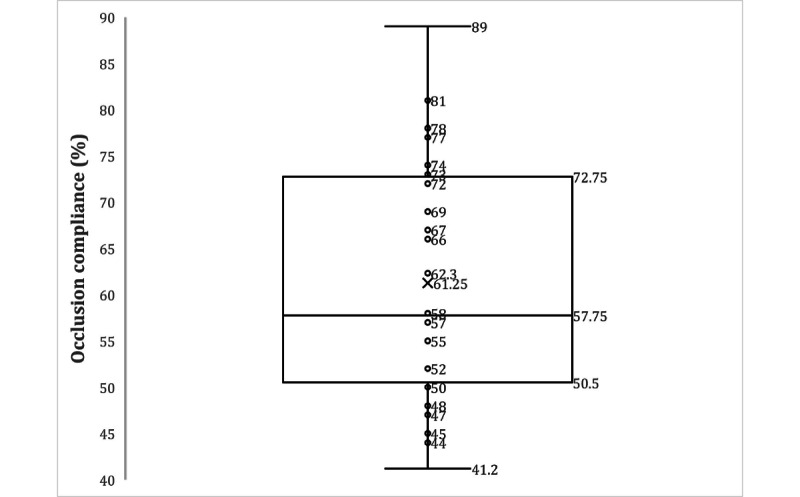
Box plot of average occlusion compliance of the meta-analysis of 24 groups from Wang et al 2015 [18-26].

Recently, a new modified version of FAVAS (2020) focused on user-friendliness of touch screen tablet computers, gamification, and attention-binding aspects, which could potentially improve patient adherence. Besides technical updates, later versions of commercially available treatment games could specifically improve engagement. We know from the literature that adherence with prescribed occlusion therapy is 62% (Table 1, Figure 1). However, in our study, we measured adherence to prescribed FAVAS therapy. The goal of this study is to evaluate whether an improved gamification aspect as well as tablet computer functionality of FAVAS therapeutic games would result in higher patient adherence compared to the earlier version. Therefore, we analyzed the electronically recorded data from a commercially available FAVAS system (Caterna Vision GmbH) in 4- to 16-year-old patients, and compared adherence to the earlier version of FAVAS 2015 with adherence to an updated version of FAVAS 2020.

**Table 1:** Literature overview of 8 papers measuring occlusion compliances in 24 groups with standard regimens to interventions like education, cartoons, and stickers to boost motivation in children and parents [18-26].

Studies and groups in paper				Objective compliance (%)^a^		Age group (years)
Stewart et al [20]				48		3-8
**Awan et al [22]**						
	3-h regimen			57.5		3-8
	6-h regimen			41.2		
**Loudon et al [21]**						
	**Education intervention group**			78		<4
		4-6	77			
		>6	74			
	**Control group**			57		<4
		4-6	52			
		>6	55			
**Stewart et al [23]**						
	6-h regimen			66		<4
	4-6			72		
	>6			69		
	12-h regimen			50		<4
	4-6			47		
	>6			58		
**Tjiam et al [24]**						
	Preimplementation cartoon			52		3-6
	Postimplementation			62.3		
**Tjiam et al [18]**						
	Educational cartoon group			89		3-6
	Reward sticker group			67		
	Parent leaflet group			73		
	Control group			55		
**Wallace et al [25]**				44		3-8
**Pradeep et al [26]**						
	Educational/motivational intervention group			81		3.5-8.9
	Control group			45		

^a^Mean 62.33% compliance (SD 13.31%).

## Methods

### Ethics Approval

This retrospective study adhered to the Declaration of Helsinki, was conducted at a single center, and was approved by the local ethics committee, Ethikkommission der Ärztekammer des Saarlandes (118/19, trial registration DRKS00017633). Due to the retrospective nature of this study and the pseudonymization at the source, no additional informed consent was required.

### Recruitment

We compared pseudonymized electronic user protocols showing the therapeutic game activity time of patients aged between 4 and 16 years; all patients were diagnosed with amblyopia by their ophthalmologist and treated with a combination of occlusion and a commercially available FAVAS therapy (Caterna Vision GmbH). The amblyopia was associated with anisometropia or strabismus. Patients had their current refractive correction worn for at least 16 weeks or until 2 consecutive visual acuity measurements, at least 8 weeks apart, did not improve by more than 1 logMAR line. The amblyopic eye had a BCVA from 20/40 to 20/200; the other eye had a BCVA of 20/32 or better, and the difference between the eyes was ≥3 logMAR lines. Children with proven learning disabilities, known epilepsy, other pre-existing ophthalmic conditions, or deprivation amblyopia (weak vision due to an organic cause) were excluded. All individuals had previous treatment with standard patching that was not successful.

### Treatments

All patients had full binocular correction with glasses, prescribed by their local eye doctors. For occlusion therapy, patients used standard eye patches. Every individual got a personalized occlusion rhythm of how many hours per day they had to wear the patch, depending on the visual acuity, fixation site at the fundus, age, and other findings. Each participant was provided with access to a home-based FAVAS, offered by Caterna Vision GmbH. The prescribed FAVAS game therapy was played every day for 30-45 minutes during occlusion time for 90 days. The treatment was reimbursed by the insurance companies. The patching regimen was individual and according to the guidelines provided by the German Ophthalmological Society. Lege artis- FAVAS regimen is standardized for 37.5 minutes per day and is applied in addition to the standard patching. The data about the effectivity in the literature all suggest that this time under FAVAS therapy has the best results. Therefore, this time interval was chosen and was also tested 2 times 20 minutes per day, but adherence with 1 time per day was higher [15,16]. Group 2015 contains a data set of patients who used FAVAS version 1.0 in 2015. They had to read the instructions for the games. For playing therapeutic games, only a keyboard and mouse with a fixed screen size of 15 inches were available. The FAVAS 2015 had a 1024 px max resolution; this has been an Adobe Flash limitation (Table 2). Group 2020 contains a data set of patients receiving therapy in 2020. They were able to play directly with high-resolution graphics and high usability. For playing games, not only a keyboard and mouse but also touchscreens with screens between 10 and 27 inches were available.

**Table 2:** FAVAS software differences.

FAVAS-version	2015	2020
Client	Web-based software solution supported by Plugin: Adobe Flash version 10	Web-based software solution
Browser	Usable, for example, with Microsoft Edge, Microsoft Internet Explorer 7, Mozilla Firefox ab 3.5, Google Chrome 3	Usable, for example, with Microsoft Edge, Mozilla Firefox und Google Chrome
Operating system	Windows from XP, or OS X from v10.4 or higher required	Plays a minor role
Stimulus	The stimulus is based on an Adobe Flash implementation	The stimulus is based on an HTML-5 implementation

### Modification of Attention-Binding Web-Based Games

**Figure 2:**
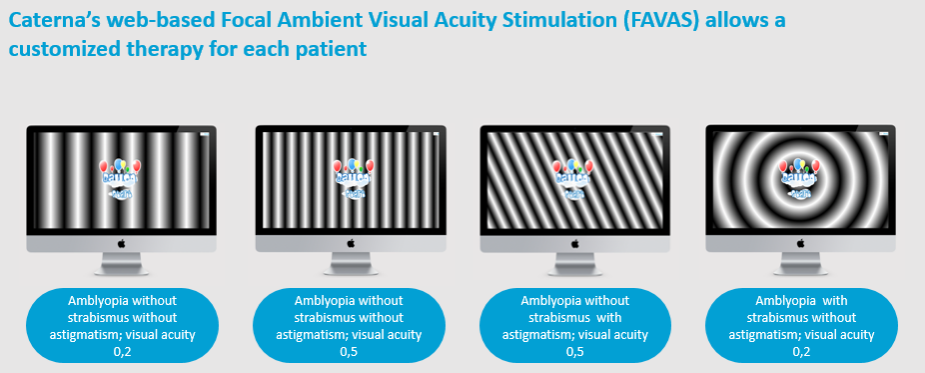
Examples of FAVAS 2020 in the customization of vertical rotating gratings according to the type of amblyopia. (A) Vertical moving gratings for anisometropic amblyopia, BCVA=0.2; (B) vertical moving gratings for anisometropic amblyopia, BCVA=0.5; (C) circular moving gratings for strabismic amblyopia, BCVA=0.2; (D) oblique moving gratings for meridional amblyopia, BCVA=0.5. BCVA: best-corrected visual acuity; FAVAS: focal ambient visual acuity stimulation.

The FAVAS 1.0 therapy was modified in a few ways: in terms of technical refinement, a larger selection with a variety of engaging games to attract children’s attention and participation was included, resulting in 9 edutainment HTML5 games for children between 4 and 16 years. Rotating gratings were personalized and selected according to the type of amblyopia (mild, moderate, or severe) with or without astigmatism and with or without strabismus. Figure 2 shows a few examples of personalized, selected FAVAS therapy. There was backward compatibility for browser, screen size, and hardware combined with better onboarding (patient manual, frequently asked questions, simplified usability). The majority of children between 4 and 16 years have access to a tablet computer, which makes access to the therapy independent of time and place. Therefore, we focused on making the therapy effective on tablet computers.

### Main Outcome Measures

The primary outcome measure was therapy adherence. Objective adherence was defined by comparing the number of minutes spent playing the computer game as monitored in the automatized, electronically recorded logbook versus the prescribed number of minutes.

### Statistical Analysis

Sample size estimates were based on data from literature reviews and participants in group 2015 pilot trials who would meet the eligibility criteria for this protocol [2,3,5,6,12]. A 2-tailed independent *t* test was used to compare continuous variables such as age and adherence between the two groups. Pearson chi-square test was used for analyzing categorical variables. *P* values smaller than .05 were considered statistically significant. Adherence over 100% (the patients played more time than prescribed) was cut down to 100% for statistical analysis. Patients in group 2015 as well as in group 2020 are patients who meet the reimbursement criteria of their health insurance. The inclusion and exclusion criteria were the same as for FAVAS 2015, so these cohorts can be carefully considered comparable. This study is a retrospective study using already collected data from patients. Due to these circumstances, a power analysis is not useful because it estimates the necessary sample size before data collection. In our case, data collection already happened, and we cannot collect more or fewer data depending on the power analysis. Following Perugini [27], we provide a sensitivity analysis instead to obtain the smallest effect which can be found using the current sample. Using a conventional power of 80%, the smallest effect which can be found is *d*=0.34 for a 2-sample *t* test. The effect size for comparison between the 2015 and 2020 group for adherence (in percent) is *d*=0.44. Thus, the empirical effect size is far beyond the minimal effect size which can be detected using the current sample and the current sample size is deemed to be sufficient. Adherence is expressed as a percentage (exercise minutes/maximum minutes).

## Results

### Overview

**Figure 3:**
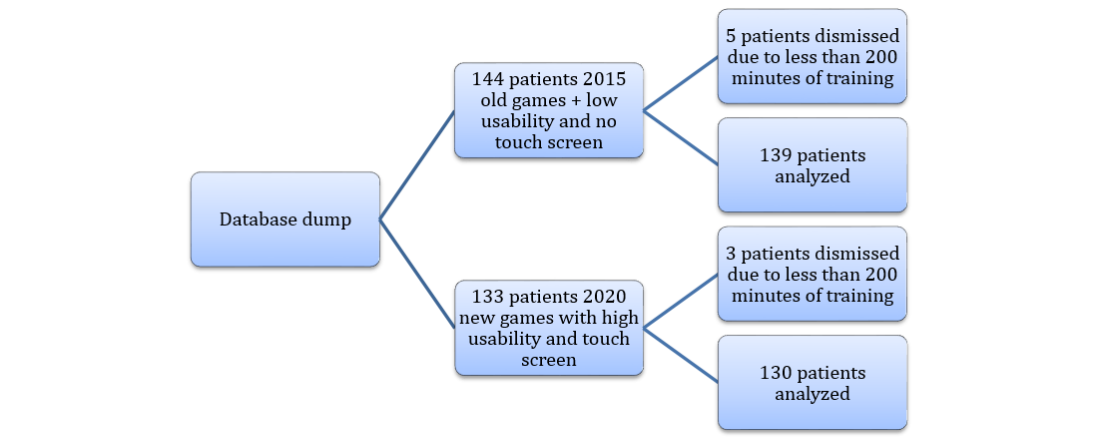
Patient flowchart, with group 2015 having 6 dropouts and group 2020 having 4 dropouts due to technical challenges. FAVAS: focal ambient visual acuity stimulation.

As shown in Figure 3, in group 2015, a total of 138 patients were analyzed; in group 2020, a total of 129 patients were analyzed. Basic characteristics of the 2 groups, such as age, sex, BCVA, and types of amblyopia, are shown in Table 3. The mean age was slightly younger in group 2020 than in group 2015, by approximately 0.6 years. Both groups had similar gender ratios and amblyopia type distributions. Patients who trained for less than 200 minutes were excluded. These were due to technical difficulties (ie, slow internet and computer problems). The patients with very little training were called by technicians and reported these problems.

**Table 3:** Baseline characteristics of the two groups.

		Group 2015 (n=138)	Group 2020 (n=129)	Chi-square or *t* test (*df*)	*P* value
**Sex**				0.005 (1)^a^	.93
	Female, n (%)	70 (50.7)	67 (51.9)		
	Male, n (%)	68 (49.3)	62 (48.1)		
Age (years), mean (SD; range)		7.8 (2.1; 4.2-15.6)	7.2 (2.3; 4.3-14.7)	2.22 (265)^b^	.03
**Amblyopia types**				0.00092^a^	.10
	Strabismic, n (%)	65 (47.1)	61 (47.3)		
	Anisometropic, n (%)	73 (52.9)	68 (52.7)		
**BCVA^c^**				N/A^d^	N/A
	<0.39	38	29		
	0.4-0.69	69	68		
	>0.7	31	32		

^a^Chi-square test.

^b^*t* test.

^c^BCVA: best-corrected visual acuity

^d^N/A: not applicable.

### Adherence

**Figure 4:**
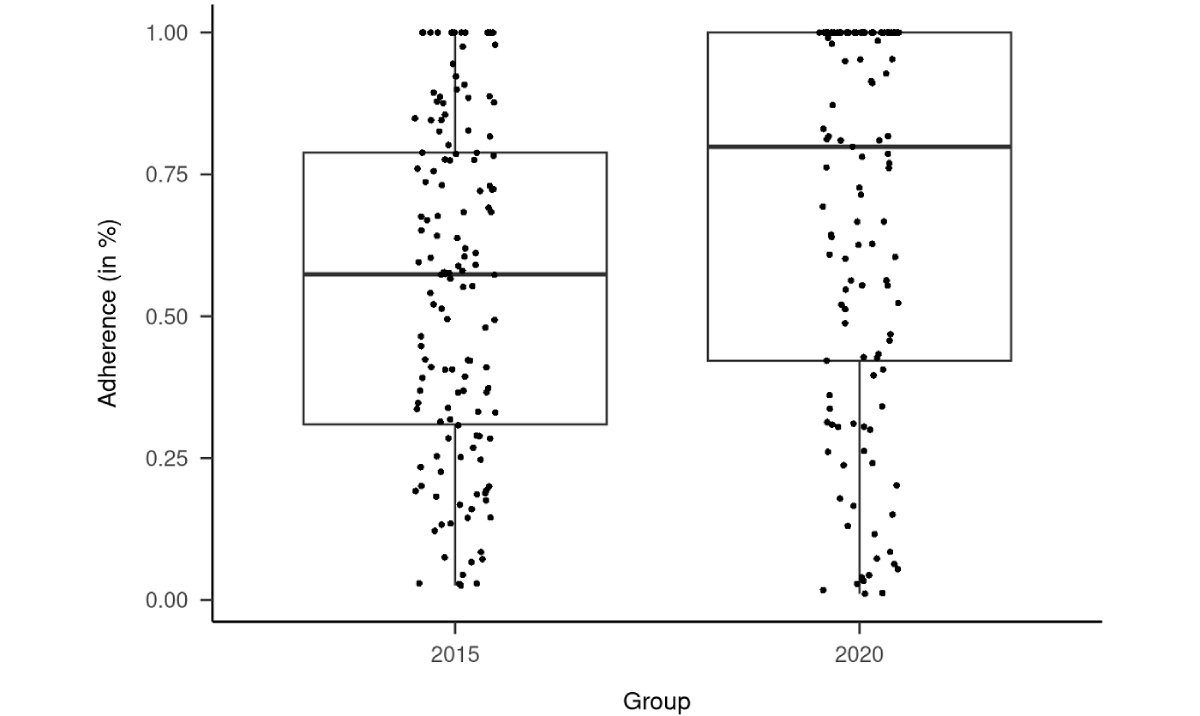
Box plot of adherence with FAVAS treatment games in group 2015 and group 2020. FAVAS: focal ambient visual acuity stimulation.

Children in group 2015 spent on average 2009.3 (SD 1372.1; range 36-5556) minutes on FAVAS games; children in group 2020 spent on average 2651.2 (SD 1557.1; range 38-5672) minutes on playing, meaning that group 2020 spent on average 641.9 minutes more time on FAVAS games than group 2015 (*t*_255.49_=3.56, *P*<.001, *d*=0.45; 95% CI 0.69-0.20). In both groups, some patients played longer than 1.5 times of the prescribed time, which indicates that some individuals enjoyed FAVAS treatment (Figure 4). In group 2015, the mean adherence was 55.0% (SD 29.4%) of the prescribed exercise time. Adherence in group 2020 significantly improved up to 68.5% (SD 33.7%) compared to group 2015 (*t*_254.38_=3.48, *P*=.001, *d*=0.44; 95% CI 0.68-0.19).

## Discussion

### Comparison With Prior Work

It was shown in earlier studies that adherence to patching is rather low, at only 60% (Table 1 and Figure 1) [2,3,5]. An overview of therapy adherence studies is shown in Table 1 and an average of occlusion compliance is shown in Figure 1. However, Loudon et al [21] and Stewart et al [20] included children younger than 4 years old, which is different from our participants. Manh et al [28] found a poor adherence of participants aged 13 to <17 years old to binocular video game treatment using an iPad at home: only 13% completed more than 75% of the prescribed 1 hour per day treatment. In this study, we showed that improving the gaming aspect and usability of FAVAS therapy can enhance patient adherence, which could possibly improve the overall therapeutic effect. Our data suggest that the new version of FAVAS with a larger selection of games attracts active participation. Backward compatibility for browsers, screen size, and hardware combined with better onboarding (patient manual, FAQ, simplified usability) seems to be a good strategy to improve patient adherence. Previous studies showed that interventions such as education cartoons for children, education flyers for parents, and sticker games have been effective in improving compliance with patching [18]. To the best of our knowledge, this is the first study on the new version of FAVAS web-based treatment games for improving adherence to patching amblyopia treatment. The modifications in this FAVAS therapy could possibly be the reason for better adherence. The reported FAVAS therapy adherence in this study was better (80%) compared to the FAVAS 2015 group, indicating that this might be beneficial for overall treatment adherence, especially for patients with low motivation for patching. However, our data do not give precise information about the rest of the prescribed occlusion time; therefore, this conclusion should be regarded with caution.

The Pediatric Eye Disease Investigator Group concluded that performing common near activities in children from 3 to 7 years old did not improve visual acuity outcomes when treating anisometropic, strabismic, or combined amblyopia with 2 hours of daily patching. The study included near activity tasks that required hand-eye coordination, such as crafts, reading, writing, and computer or video games [13]. These near tasks cannot be compared to the FAVAS game. FAVAS differs in several ways from the known moving grating stimulation Cambridge Stimulator (CAM) treatment. CAM used high-contrast square-wave gratings, which were rotated in front of the amblyopic eye while playing on a transparent cover in front of the stimulator. It was initially reported to improve outcomes when combined with patching but failed to succeed in subsequent prospective randomized controlled studies [29,30]. Beyond CAM treatment, FAVAS relies not only on the spatial frequency selectivity of the ambient background stimulus but also on the interaction of its coordinated temporal frequency parameters with the focal sensory-motoric gaming activity (Kämpf et al) [15-17]. While CAM treatment is a passive treatment, FAVAS is an active treatment because patients have to interact with games. Interactions with FAVAS games require eye-hand coordination. The treatment duration differs. CAM treatment was applied for only 7 minutes, while FAVAS was applied for 30 to 45 minutes for 90 days in this study. During interactive binocular treatment, the participant wears shutter glasses, and the images are presented to both eyes, but parts of the image are presented only to the amblyopic eye. A fine and movable stimulus is presented to the amblyopic eye, and fixed targets or backgrounds are presented to the dominant eye. Additionally, half of 1 image for each eye is shown simultaneously, and identical images are demonstrated for both eyes with a small retinal disparity [31].

It has been proven before that monocular training improves visual acuity, but contrast sensitivity improves more when grating patterns are used [14,32]. Previous studies had positive results on stereoacuity after monocular training [33]. On the other hand, not all studies show evidence of improved visual acuity after dichoptic treatment [28-34]. It should be evaluated further regarding functional outcomes such as visual acuity, stereopsis, or contrast sensitivity improvement during FAVAS therapy. A recent study showed that near visual acuity was better than distance visual acuity in amblyopic patients, so that near visual acuity tests can be used to increase the sensitivity and specificity of the distance visual acuity tests for screening and diagnosis of amblyopia. However, other studies do not find this difference and think it is likely due to test-retest variability [35,36]. This is also an aspect we have to take into account.

### Limitations

Our study has a few limitations. One is that the retrospective character and the fact that data about patching duration were only measured using electronic logbooks during the therapeutic game activity should be regarded critically. During ongoing therapeutic interventions, the adherence to patching is, on average, continuously decreasing the longer the treatment lasts [19]. Thus, our future tasks will not only be increasing average adherence but also changing the current dynamic of adherence by slowing down, maintaining, or reversing the decreasing adherence trend with computer-assisted therapeutic interventions. The results of this study show an attractive option: improving gamification and adding tablet computer functionality increased adherence, which might stop a negative dynamic during a long therapy. However, further studies with automatic occlusion dose monitoring should be added to verify our findings [37]. The long-term compliance of the FAVAS therapy has to be investigated since the binocular treatment of amblyopia using videogames study says that compliance is high early in therapy but begins to fall after 6 weeks if the game is not changed [13]. The strengths of this study are the fact that the data were generated not in an artificial setting of a trial but reflect clinical reality; also, participation was monitored objectively through electronic log files and did not include patient questionnaires regarding adherence.

### Conclusions

FAVAS therapeutic game 2020 with an improved gamification aspect as well as tablet computer functionality increased adherence significantly compared to the earlier version of FAVAS 2015, indicating that FAVAS 2020 could be an effective approach to support adherence to amblyopia treatment.

## Abbreviations

BCVA: best-corrected visual acuity
CAM: Cambridge Stimulator
FAVAS: focal ambient visual acuity stimulation
